# Public perceptions about climate change mitigation in British Columbia's forest sector

**DOI:** 10.1371/journal.pone.0195999

**Published:** 2018-04-23

**Authors:** Guillaume Peterson St-Laurent, Shannon Hagerman, Robert Kozak, George Hoberg

**Affiliations:** 1 Institute for Resources, Environment and Sustainability, University of British Columbia, Vancouver, British Columbia, Canada; 2 Faculty of Forestry, University of British Columbia, Vancouver, British Columbia, Canada; 3 Liu Institute for Global Issues, University of British Columbia, Vancouver, British Columbia, Canada; Chinese Academy of Forestry, CHINA

## Abstract

The role of forest management in mitigating climate change is a central concern for the Canadian province of British Columbia. The successful implementation of forest management activities to achieve climate change mitigation in British Columbia will be strongly influenced by public support or opposition. While we now have increasingly clear ideas of the management opportunities associated with forest mitigation and some insight into public support for climate change mitigation in the context of sustainable forest management, very little is known with respect to the levels and basis of public support for potential forest management strategies to mitigate climate change. This paper, by describing the results of a web-based survey, documents levels of public support for the implementation of eight forest carbon mitigation strategies in British Columbia’s forest sector, and examines and quantifies the influence of the factors that shape this support. Overall, respondents ascribed a high level of importance to forest carbon mitigation and supported all of the eight proposed strategies, indicating that the British Columbia public is inclined to consider alternative practices in managing forests and wood products to mitigate climate change. That said, we found differences in levels of support for the mitigation strategies. In general, we found greater levels of support for a rehabilitation strategy (e.g. reforestation of unproductive forest land), and to a lesser extent for conservation strategies (e.g. old growth conservation, reduced harvest) over enhanced forest management strategies (e.g. improved harvesting and silvicultural techniques). We also highlighted multiple variables within the British Columbia population that appear to play a role in predicting levels of support for conservation and/or enhanced forest management strategies, including environmental values, risk perception, trust in groups of actors, prioritized objectives of forest management and socio-demographic factors.

## 1 Introduction

In recent years, the upsurge of attention to climate change has placed forests and their central role in the carbon cycle at the forefront of attention. The management of forest ecosystems has the potential to reduce greenhouse gases emissions and/or increase carbon removals from the atmosphere [1, 2]. It is also important to consider mitigation opportunities from increasing the carbon stored in wood products [3, 4] and increasing substitution of wood products for other products and fossil fuels whose production and use cause more greenhouse gases emissions on a life-cycle basis [5]. Consequently, policy makers and forest managers are now increasingly considering forest carbon as another value to be managed in the forests in addition to other benefits such as timber supply, employment, biodiversity and water conservation.

As for many other jurisdictions, the role of forest management in mitigating climate change is a central concern for the Canadian province of British Columbia (BC), where more than 60% of the territory’s 95 million hectares is forested [6, 7]. The province’s recently announced Climate Leadership Plan indicates that “we can harness this opportunity to sequester atmospheric carbon dioxide in this tremendous public asset [forests] through intensive forest management practices and storing carbon in long-lived wood products” [6]. At the same time, a recent study identified a number of barriers to the implementation of carbon mitigation policies in BC forests (e.g. forest carbon management not explicitly addressed in forest and climate policies) and concluded that BC has thus far enacted very few forest carbon mitigation policies in practice [8].

Designing mitigation options for BC involving forests will require assessment of management alternatives that are informed by scientific understanding (e.g. carbon modelling) and an in-depth understanding of policy gaps (e.g., modest coverage of forest carbon in existing policies). However, the successful implementation of forest management to achieve climate change mitigation will also be strongly influenced by public support or opposition. The acceptance of new resource management and climate policies often rely on the need for the general public to understand, accept and perceive them as effective, fair and legitimate [9, 10]. This is particularly the case for BC’s forests, which have been since the 1980’s at the center of disputes over their management and conservation (e.g., Clayoquot Sound, Great Bear Rainforest [11, 12]). Furthermore, the recent purchase by the provincial government of carbon credits originating from controversial BC-based forest carbon offset projects has already been publicly criticized, thereby attracting media attention and making forest carbon management a contentious issue [13].

A previous survey evaluating the prioritization of values of six forest-dependent communities in BC in the context of sustainable forest management found high recognition of the importance of reducing climate change [14]. However, no study to date has explored public opinion regarding forest management strategies specifically designed for their potential to mitigate climate change and the factors that influence their acceptance. Thus, while we now have increasingly clear ideas of the management opportunities associated with forest mitigation in British Columbia [7, 15] and in Canada [3, 16–18], and some insight into public support for climate change mitigation in the context of sustainable forest management [14], very little is known with respect to the levels and basis of public support for potential forest management strategies to mitigate climate change. This paper is thus the first to examine and document the level of support in the BC population at large and examine how demographic characteristics as well as explanatory variables derived from the literature on cognitive and value-based dimensions of risk perception (i.e., environmental values, knowledge, trust in decision-makers, perceived climate risks and preferred objectives of forest management) influence support for different types of mitigation strategies.

## 2 Policy background: Forest carbon mitigation in BC

Forest ecosystems and products made from wood comprise various reservoirs that store, capture or release carbon. Activities that reduce emissions or increase removals compared to business-as-usual or “baseline” levels are considered climate change mitigation actions. First, climate change mitigation can be achieved through the conservation of existing or the creation of new forest carbon sinks through avoided deforestation (i.e., the permanent removal of forest and change to non-forest land uses) and afforestation (i.e. the creation of new forest where none has existed for some time). However, because deforestation rates in BC are comparatively low, especially in relation to tropical regions [19, 20], the opportunities associated with avoided deforestation and afforestation [21] are scarce in BC.

Second, forest carbon density, referring to the amount of carbon per hectare of forest, can be maintained or increased through forest management strategies. Such actions involve the trade-offs between: (1) natural forest conservation and reduced harvest strategies and (2) forest harvesting and sustainable forest management. Conservation strategies provide potential mitigation benefits because natural forests typically store more carbon than managed forests because of their longer disturbance cycles and greater proportion of older stands [22, 23]. In contrast, strategies focused on timber harvesting and more intensive forest management offer mitigation potential associated with increased forest carbon uptake rates (e.g., genetically improved seeds), improved harvesting techniques (e.g., avoided slashburning, maximized utilization at harvest) and enhanced production of long-lived wood products, which in turn can increase substitution benefits [3]. The frequency of occurrence of natural disturbances represents an important factor to consider when defining such forest management strategies [23]. While conservation practices that maintain carbon stocks will be challenging in areas facing frequent natural disturbances, such as BC’s boreal and interior forests [24], better opportunities are encountered in ecosystems characterized by infrequent natural disturbance patterns and high carbon density, such as the coastal temperate rainforest [22].

Third, strategies that focus on the use of wood products can also be effective at increasing carbon removals and reducing greenhouse gas emissions to the atmosphere. To analyse the impact of wood products on atmospheric carbon, one has to evaluate a product’s whole life cycle, from extraction to end-of-life management and potentially beyond [3, 25]. In particular, the time over which carbon is stored in wood products depends greatly on their life duration. Some wood products have very short useful half lives, such as paper (2.5 years), whereas others have longer-term carbon storage potential such as the lumber encountered in single family homes (>90 year half-life) and commercial buildings (>75 years) [26, 27]. Wood products can also be used as substitute for other products, so as to offset emissions from more energy-intensive products such as concrete and steel (i.e., material substitution) or fossil fuels such as coal and natural gas (i.e., energy substitution) [28, 29].

## 3 Hypotheses development

While public opinion on forest carbon mitigation has not been widely documented thus far, insights from the literature on cognitive and value-based dimensions of environmental, forest management and climate risk perception more broadly can provide ideas as to which variables are likely to influence public preference for specific forest management strategies in the context of climate change. The next section provides the theoretical basis and motivations for our survey design. In particular, we present a brief synthesis of key insights from relevant literatures to justify the set of variables that were selected for this study.

### 3.1 Environmental values

Forests in Canada and in BC, as in many other parts of the world, have historically been managed for the purpose of a primary objective, namely timber production [30]. However, forests are increasingly being managed based on multiple public values [31, 32]. In particular, over the past three decades, an increase in interest in environmental values has led to a context where “societal worldviews fundamentally have shifted from a paradigm of subservience of nature and the environment to meet human needs to a more holistic paradigm whereby nature is perceived as having a right to exist for its own sake, regardless of its usefulness to humankind” [33].

Because of the important influence of values on preferences for environmental and risk management scenarios, numerous scales have been created and used for the purpose of evaluating public environmental values [34], including the New Environmental Paradigm [35, 36] and the cultural theory scale [37]. In the context of forest management, values can be defined as “an individual’s orientation toward forests” [38]. They are often situated somewhere on a continuum ranging from anthropocentric to biocentric, as observed in the Forest Value Scale first developed by Steel et al. [39] and regularly used since [33, 40–42].

An anthropocentric value-orientation is associated with the perception that nature and forests represent a resource to be utilized by humans for their well-being; it mainly focuses on humans and their needs [39]. As Aubin et al. [43] explain, anthropocentric-oriented individuals generally believe that management practices that are informed by science and technology have the potential to improve forests and their productivity. In contrast, a biocentric value orientation is natured-centered, elevating “the requirements and values of all natural organisms, species, and ecosystems to center stage and, in some versions, makes the earth or nature as a whole the focus of ‘moral considerability’” [39]. While still recognizing the importance of humans, biocentrists locate them within the greater natural context and perceive nature as holding intrinsic value on its own. As such, humans have the responsibility to protect nature: “A thing is right when it tends to preserve the integrity, stability, and beauty of the biotic community. It is wrong when it tends otherwise” [44].

In the context of forest management and climate change, a biocentric orientation would normally encourage practices that limit human impact on nature, whereas anthropocentric individuals would not hesitate to draw on technological solutions and the large-scale implementation or intensification of forest management strategies if it had the potential to lead to significant mitigation potential. However, the emerging discussions about climate and atmospheric changes and their increasingly important impacts on ecosystems somewhat complicates the anthropocentric versus biocentric views. In fact, climate change mitigation is about limiting the human impact on climate and hence on ecosystems in the long run. Biocentric individuals may thus justify active intervention in forests, for instance when inaction may have more damaging impacts on ecosystems than active forest management.

### 3.2 Risk perception of climate change

A fundamental assumption encountered in the literature is that individuals who perceive environmental impacts as threatening will be more willing to take actions to mitigate their risks [45, 46]. This notion is at the forefront of discussions around climate change, leading to the belief that risk perception is an important determinant of the public’s willingness to take actions to mitigate climate change [47–49].

Research in this domain has shown that the public generally perceives the threats posed by climate change as less important than the scientists do, especially in developed countries [47, 50, 51]. This observation is linked to the vague and seemingly distant features of climate change, both spatially and temporally. Consequently, studies increasingly highlight the importance of personal experience in both shaping risk perception and willingness to act in mitigating climate change [52–54]. In effect, the likelihood that climate change is perceived to pose a serious risk generally increases when someone has recently experienced an event that they directly associate with climate change [47].

### 3.3 Knowledge of climate change and forest management

The differences encountered between experts and the public in their knowledge of environmental issues such as forest management and climate change is often identified as one of the main reasons explaining why they have different perceptions of risk [55]. However, while some studies do not find significant relationships between knowledge and risk perception [56], others even contradict this assumption by showing that higher knowledge can actually reduce risk perception of climate change [57, 58] or of forest pest outbreaks [59].

At the same time, recent studies have also shown that a large proportion of the world’s population does not believe that climate change is human-based [60, 61], leading, in turn, to lower risk perception [62]. Furthermore, as Lorenzi and Pidgeon [60] explain, a misunderstanding of the human-induced causes of climate change “may be creating a false impression that activities which lead to dangerous outcomes are in fact safe”.

### 3.4 Trust in experts, decision-makers and other influential actors

Trust in scientists and experts, including government agencies and environmental groups, is often presented as being instrumental in understanding risk perception of climate change [63, 64]. Nevertheless, divergent trends have been observed. On the one hand, perceived risk might increase if members of the public realize that experts are worrying about the negative impacts of climate change [65]. On the other hand, Kellstedt et al. [57] found that trust in scientists can increase the confidence that technical solutions will be found, thereby reducing perceived risk.

In Canada, a high confidence in forest agencies, experts and industry has been positively associated with acceptance of forest management policies and their implied risks [55, 66]. Olsen and Shindler [67] explain, using the case study of forest fire management, that trust in forest agencies and land managers is particularly important when the public lacks familiarity with, but values the outcomes of management practices. In contrast, trust in environmental groups and First Nations, which are habitually associated in British Columbia with forest conservation [68–70], generally lead to greater support for conservation practices.

### 3.5 Objectives of forest management as expression of values

An individual’s prioritized objectives of forest management can also contribute to explaining whether or not he or she will support or oppose different practices. As Aubin et al. [43] explain, referring to a case study of climate change adaptation in Canada’s forests, “the debate has moved beyond a strict scientific discussion into the arena of beliefs, values, visions of the future, and subjective perceptions of risk and desirable outcomes”. In the context of climate change mitigation in forests, objectives can be defined as what really matters when thinking about forest carbon mitigation; they represent what is prioritized by each individual [71]. An objective often represents an expression of citizens’ beliefs and values [72, 73]. For instance, an individual who prioritizes biodiversity conservation will most likely support conservation-based mitigation strategies. On the contrary, individuals focusing on economic productivity of forests will presumably support mitigation strategies oriented towards economic development and timber productivity.

### 3.6 Hypothesized effect of explanatory variables on support for forest carbon mitigation strategies

Table 1 summarizes the expected directionality of the variables’ effect on levels of support for forest carbon management strategies, as identified in our literature review of the cognitive and value-based dimensions of risk perception. Because the possible strategies offer variation in terms of their levels of human intervention, we made a distinction between strategies with relatively less (e.g., conservation, reduction in harvest) and more human intervention (e.g., improved commercial harvesting techniques).

**Table 1:** Expected directionality of the explanatory variables’ effect on levels of support for forest carbon mitigation strategies based on their relative levels of human-intervention. A positive sign indicates a positive relationship, a negative sign indicates a negative relationship and a positive/negative sign indicates that the relationship could either be positive or negative.

Explanatory variable	Directionality	
	Less human intervention	More human intervention
**Environmental values**		
- Anthropocentric orientation	+/-	+
- Biocentric orientation	+	+/-
**Risk perception of climate change**		
- Belief on the human-cause of climate change	+	+
- Perceived risk of climate change	+	+
- Personal experience with climate change	+	+
**Knowledge**		
- Climate change and forest management	+/-	+/-
**Trust in different actors**		
- Experts	+/-	+
- Environmental groups and First Nation leaders	+	+/-
**Prioritized outcomes**		
- Economic and social	-	+
- Environmental	+	+/-

## 4 Methods

### 4.1 Web-based survey

A questionnaire was used to evaluate levels of public support for the implementation of possible forest carbon mitigation strategies in BC’s forests, and to examine and quantify the influence of the factors that shape this support. The questionnaire comprised 47 questions, including rating scales, ranking questions and open-ended questions if respondents wanted to provide additional comments (see S1 Appendix for detailed description of all questions). Two types of rating scales were used: 1) ordinal scales, where no attempt was made to combine individual responses, and 2) interval scales, where the scores of each individual response were combined into one or multiple composite scales [74]. The study was approved by the University of British Columbia (UBC) Behavioral Research Ethics Board (ethics certificate number H15-01354). Participants were provided with a consent form at the beginning of the online survey. Submission of the questionnaire confirmed agreement to participate in the research study.

We used FluidSurveys (http://fluidsurveys.com) to create an online survey that was distributed to BC’s general public by a digital data collection company (ResearchNow, https://www.researchnow.com). While online surveys provide various advantages, they also face limitations that are worth noting [75], including limited sampling availability (i.e., certain individuals are less likely to complete online surveys) and the inability to clarify respondents’ questions and misunderstandings, which could be a concern when dealing with complex issues such as forest carbon management.

To ensure a representative sample of the general population, we programmed quotas based on the latest population census (2011) in terms of age (19–34 years: 27%; and 35–54 years: 36%), gender (Female: 52%) and proportion of the population living in the two major metropolitan areas (Vancouver and Victoria: 69%). These quotas avoided an overrepresentation of certain segments of the population (e.g., living in urban centres, younger individuals more apt to respond to online survey) within the panel sample. We collected a total of 1484 completed surveys (90% completion rate) between January 23 and 30, 2017. This total excludes surveys filled by respondents who do not believe that climate change is happening (n = 16, 1.07% of total respondents). We chose to exclude these respondents because we considered that people who do not believe climate change is happening, notwithstanding its cause, would not be interested in completing a survey about climate change mitigation strategies. In any case, the very small sample size indicates that their inclusion would have had very limited impact on the results. We also excluded surveys that were completed in less than five and a half minutes (n = 86), the minimum time that we judged necessary to accurately complete the survey during pre-testing.

#### 4.1.1 Dependent variables

In the questionnaire, we provided respondents with short descriptions of eight possible strategies to mitigate climate change in BC’s forest sector (hereafter “mitigation strategies”) and their potential climate mitigation benefits (Table 2). We simplified the descriptions of the strategies as much as possible, a result of multiple iterations and comments from pilot testing and reviews of the questionnaire. However, because of the complexity of the topic, we acknowledge that the description of the strategies may have proven difficult to understand for certain individuals with limited knowledge of forest management.

**Table 2:** Description of the eight forest carbon mitigation strategies.

*1*. *Bioenergy strategy*: Produce bioenergy with residues that are not currently collected during commercial harvesting (for example, branches, tree tops, and unusable trees), and which would otherwise decay in the forest.
*Mitigation Benefits*: Use of bioenergy instead of fossil fuels (for example, coal, natural gas) whose production and use generate more net greenhouse gas emissions and climate change.
*2*. *Harvest efficiency strategy*: Improve harvest efficiency by collecting more wood per hectare harvested commercially and using it for products, thereby reducing the amount of harvest waste that is left on the forest floor to decay or be burned, and reducing the total area harvested while keeping the total harvest volume unchanged.
*Mitigation benefit*: Reduce greenhouse gas emissions from decay or burning of wood and (2) reduce the area harvested.
*3*. *Increased growth rate strategy*: Increase the growth rate of trees above current levels through various techniques (e.g., planting improved seeds or tree species, fertilization).
*Mitigation benefit*: Capture carbon from the atmosphere more rapidly because trees grow faster.
*4*. *Increased harvest strategy*: Increase the forest areas that can be harvested commercially, focusing on areas most likely to be affected by insects and fires.
*Mitigation benefit*: Increase carbon storage by producing more wood products that can be used in place of products like cement and steel whose production and use generate more greenhouse gas emissions and climate change.
*5*. *Longer-lived wood products strategy*: Increase the production of longer-lived wood products like lumber and wood panels for use in construction, and correspondingly decrease the production of shorter-lived products like paper.
*Mitigation benefit*: (1) Increase the time over which carbon is stored in wood products and (2) use the wood products instead of products like cement and steel whose production and use generate more greenhouse gas emissions and climate change.
*6*. *Old growth conservation strategy*: Prevent commercial harvesting in old growth forests.
*Mitigation benefit*: Protect old growth forests with high quantities of carbon.
*7*. *Reduced harvest strategy*: Reduce the area of forest that can be harvested commercially each year.
*Mitigation benefit*: Avoid emissions by protecting existing carbon found in managed forests.
*8*. *Rehabilitation strategy*: Plant trees in areas recently affected by insects and fires where trees are growing poorly.
*Mitigation benefit*: (1) Reduce greenhouse gas emissions from potential decay and wildfires and (2) capture carbon from the atmosphere more rapidly because trees are healthier and grow faster.

Respondents identified their levels of support for the mitigation strategies using five-point interval scale questions, which were combined into composite scales using a factor analysis. We used the level of support for the mitigation strategies as the dependent variables in the main analysis (i.e., multiple regressions, see analysis section below). Two strategies (i.e., bioenergy and longer-lived wood products) rely on the use of wood products for either carbon storage and material substitution or energy substitution. The remaining six strategies focus on forest management. We did not select any strategy that increases or maintains forest areas (i.e., land-use changes such as reduced deforestation and increased afforestation) because of their relatively low mitigation potential in BC. The strategies purposefully offer variation in terms of their expected levels of human intervention in the forests.

#### 4.1.2 Independent variables

Based on the literature review, we assessed the influence of various independent variables on the levels of support for the different strategies (complete questions can be found in S1 Appendix). First, respondents rated seven questions on their levels of concerns towards, and expected harm from, climate change using five-point interval scale questions. An aggregated average index on *risk perception of climate change* was assigned to each respondent. In order to test the relationship between risk perception and willingness to act, and to evaluate if forest carbon mitigation is equally valued compared to other sectors, we also had respondents, using five-point ordinal scale questions, identify how important they think it is to deal with climate change mitigation in BC both: 1) across all sectors and 2) in the forest sector specifically. We also asked respondents to indicate how many times they experienced natural disasters or extreme weather events that they associated with climate change in the last 10 years.

Second, we evaluated respondents belief on the *cause of climate change* (i.e., humans and/or natural processes; a question adapted from Leiserowitz et al. [61]). We used this question to exclude from the analysis the respondents who believe that climate change is not happening (n = 16). Furthermore, we assessed the respondents’ levels of *knowledge of climate change in the context of forests and their management* by having them rate their knowledge of four topics related to forest management in the context of climate change on five-point interval scale questions. While self-reported knowledge is sometimes criticized for inaccurately representing the actual knowledge of survey participants [76], it is still widely used and was shown to be as effective as cross-examination with true or false questions [42]. We chose to use self-reported knowledge over other options (i.e., quiz with true/false questions) to limit the length of the survey, but we acknowledge the potential limitations of this strategy.

Third, we used the Forest Values scale [39] to assess *environmental value orientation*. We asked respondents rate their levels of agreement on eight statements, four associated with anthropocentric beliefs and four with biocentric beliefs, using a five-point interval scale.

Fourth, we had respondents rate their *levels of trust* towards seven actors (scientists, forest industry, BC’s provincial government, Canadian federal government, environmental groups, professional foresters, and First Nations leaders) when it comes to providing information on climate change issues in BC’s forests using a five-point interval scale.

Fifth, respondents were asked to rank the importance (from the most to the least) of five *objectives when selecting forest management strategies* to mitigate climate change in BC’s forests. These objectives were: 1) costs; 2) effectiveness at mitigating climate change (mitigation); 3) impacts on biodiversity; 4) effectiveness at reducing natural disturbances (adaptation); and 5) economic impacts for local communities (local economy).

Finally, various sociodemographic measures were collected, including gender, age, income, education, employment in the forest sector, support for political party (transformed into conservative/progressive political ideologies) and residence type (i.e., rural/suburban/urban). All of the demographic variables were used in the analysis, except for income because of a low response rate to this question.

### 4.2 Data analysis

As previously explained, data from rating scales that were used individually (i.e., no attempt was made to combine them into composite scales) were assumed to be ordinal in nature and, therefore, warranted the use of nonparametric statistical analyses. In contrast, the composite scales created from grouping rating scale questions were assumed to be interval in nature, meaning that we were able to analyse them with parametric statistics [74]. Both dependent and independent variables were analysed with descriptive statistics (means, medians, frequencies) and inferential statistics best adapted either for ordinal (i.e., Mann–Whitney U test, Spearman Rho) or interval data (Student’s t-test, Pearson's r).

We carried out individual factor analyses with varimax rotations with each independent variable (i.e., environmental value, knowledge and trust in different actors) to group interval scale questions into factors when applicable. The dependent variables (i.e., level of support for each strategy) were also factor analysed to cluster the strategies into a reduced set of categories. In each case, factors were extracted until the eigenvalue fell below 1. A minimum loading of 0.40 was used to identify which items belong to any given factor. New indexes of interval data were created by averaging the scores of each statement that loaded on the factors. A Cronbach’s alpha reliability coefficient (α) was also computed to evaluate the internal consistency of the new scales.

Multiple linear regressions were carried out to evaluate the contribution of the independent variables to the dependent variables (i.e., level of support for the clusters of strategies identified with the factor analysis). The explained variance (Adjusted R^2^ value), which represents the proportion of the variation in the independent variable that can be explained by the regression model, was used to illustrate the relative contribution of the block of independent variables to the level of support for the clustered strategies.

## 5 Results and discussion

Before exploring the results of the multiple regressions and their implications in detail, we summarize both the independent explanatory variables and the levels of support for the different mitigation strategies (i.e., dependent variables). An overview of respondents’ demographic data can be found in S2 Appendix. We also present the results of a factor analysis that, by identifying interdependencies between levels of support for the different mitigation strategies, allowed us to cluster them into two underlying categories.

### 5.1 Explanatory variables for basis of support

Approximately half of the respondents (n = 735) believed that climate change is mostly caused by human activities. This finding is similar to the 52% found in a 2015 study in the United States [61] and higher than a recent estimate for BC’s population (42%) [77]. The rest of our respondents either indicated that climate change was caused equally by natural changes in the environment and human activities (40.5%; n = 601), was caused mostly by natural changes in the environment (7.9%; n = 117) or that it was not happening (1.1%; n = 16; excluded from the analysis). Accordingly, a large proportion of our respondents believe that climate change is mostly or partly caused by human activities (90.5%), which is considerably higher than in the United States (57%) [61] or the estimate recently calculated for BC (62%) [77]. For the regression analysis, each of the respondents was assigned a binary variable to identify who believes and who does not believe that climate change is caused mostly by human activities.

Around half of the respondents (50.9%; n = 756) indicated having experienced at least one natural event which they attribute to climate change in the last 10 years. This finding is high compared to previous studies in North America [53, 61], which found levels of between 25% and 35%. While we did not ask respondents to specify what type of natural event they experienced, comments in the open-ended portion of the questionnaires indicate that many respondents referred to the mountain pine beetle epidemic that has affected BC’s interior regions since the early 2000s [46, 78] or the recent forest fire events observed in various regions across BC (e.g., Kelowna, Lower Mainland [79, 80]). A binary variable was created to identify respondents who had experienced at least one event that they associated with climate change.

The survey results also highlighted a high perceived level of importance on 5-point ordinal scale questions for dealing with climate change mitigation in BC’s forest sector (mean = 4.00, standard deviation = 0.90) and across all sectors (mean = 3.99, standard deviation = 0.87; Mann–Whitney U = 1,022,500, p = 0.60). We found a strong correlation between respondents’ perceived risk of climate change (mean = 3.39, standard deviation = 0.82, α = 0.94) and perceived importance of climate change mitigation in the forest sector (Spearman's rho = 0.55, p<0.001), confirming the widely-accepted notion that risk perception and willingness to act are correlated [47–49]. Because of this strong correlation, we used only the risk perception index in the regression analysis.

**Fig 1:**
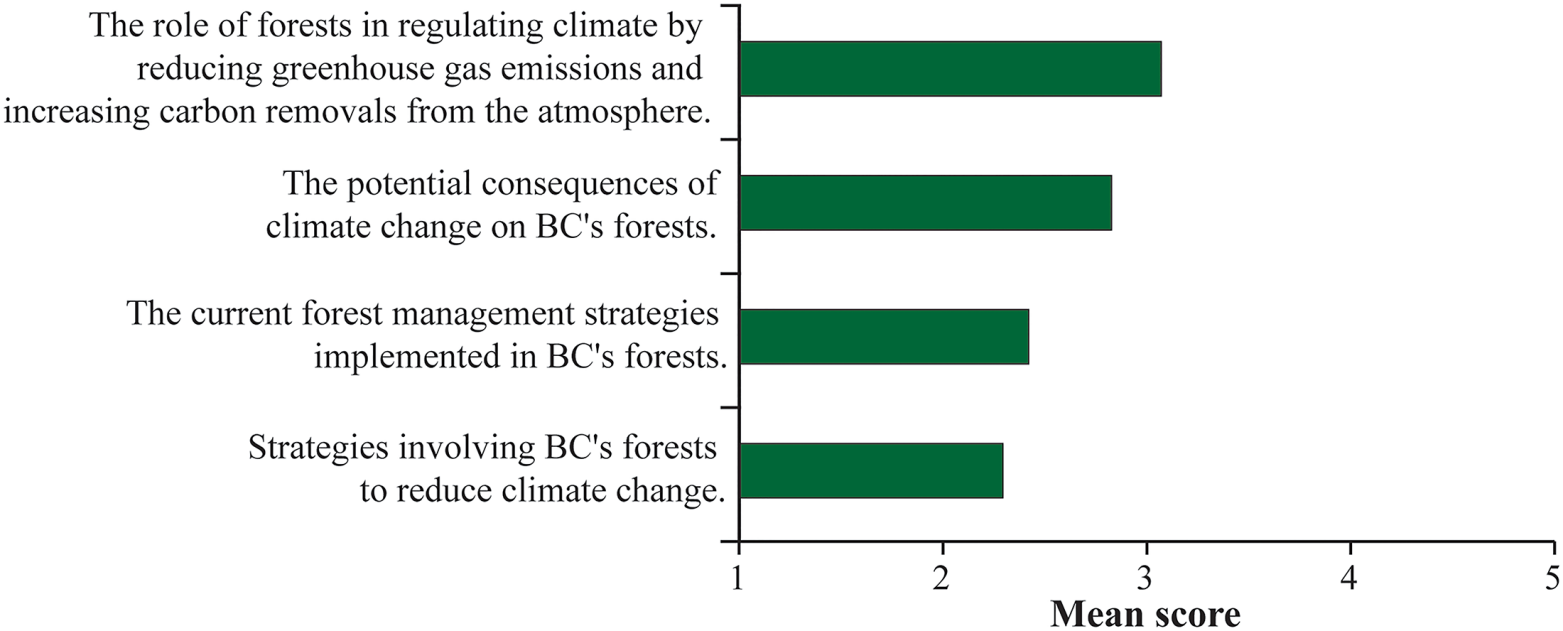
Mean scores representing the degree of knowledge about four different topics related to climate change in the context of forests and their management, with 1 = not at all knowledgeable and 5 = very knowledgeable.

Fig 1 shows respondents’ level of knowledge with regards to four topics associated with climate change in the context of forests and their management. A factor analysis was conducted on the knowledge of the respondents on the four topics, and the results suggested a one factor solution (Table 3). Consequently, an aggregated knowledge variable was created by calculating the average of the four topics (mean = 2.65, standard deviation = 0.72, α = 0.87).

**Table 3:** Knowledge scale items and factor loadings. Factors’ loadings in bold indicate that they have been selected in a factor.

	Loading
**Factor 1** (α = 0.87)	
The role of forests in regulating climate by reducing greenhouse gas emissions and increasing carbon removals from the atmosphere.	**0.627**
The potential consequences of climate change on BC's forests.	**0.731**
The current forest management practices implemented in BC's forests.	**0.907**
Strategies involving BC's forests to reduce climate change.	**0.874**

Two factors of environmental values were extracted using a subsequent factor analysis, with latent meanings being attributed to anthropocentric and biocentric value orientations (Table 4). Each respondent was assigned two indices to be used in the regressions by averaging the score of each statement that loaded on each of the two factors. On average, respondents showed a greater biocentric (mean = 4.3, standard deviation = 0.64, α = 0.83) than anthropocentric orientation (M = 2.1, SD = 0.86, α = 0.76), with significant difference between the means of the two averages (t(2725) = -81.27, p<0.001). This result is consistent with other studies that have observed the rise in importance of environmental values and forest conservation observed in North America, generally [39, 40], and in British Columbia, more specifically [11, 14, 32].

**Table 4:** Environmental value scale items and factor loadings. Factors’ loadings in bold indicate that they have been selected in a factor.

	Loading	
**Factor 1** (Anthropocentric, α = 0.76)		
Forests should be managed to meet the needs of as many people as possible.	0.397	
The primary use of forests should be for products that are useful to humans.	**0.636**	-0.219
Forests that are not used for the benefit of humans are a waste of our natural resources.	**0.694**	-0.336
Forests are valuable only if they produce jobs and income for people.	**0.719**	-0.342
**Factor 2** (Biocentric, α = 0.83)		
Forests give us a sense of peace and well-being.	-0.109	**0.766**
Whether or not I get to visit the forest as much as I like, it is important for me to know that forests exist in BC.	-0.183	**0.704**
Forests have a right to exist for their own sake, regardless of human concerns and uses.	-0.175	**0.663**
Humans should have more love, respect and admiration for forests.	-0.136	**0.769**

**Fig 2:**
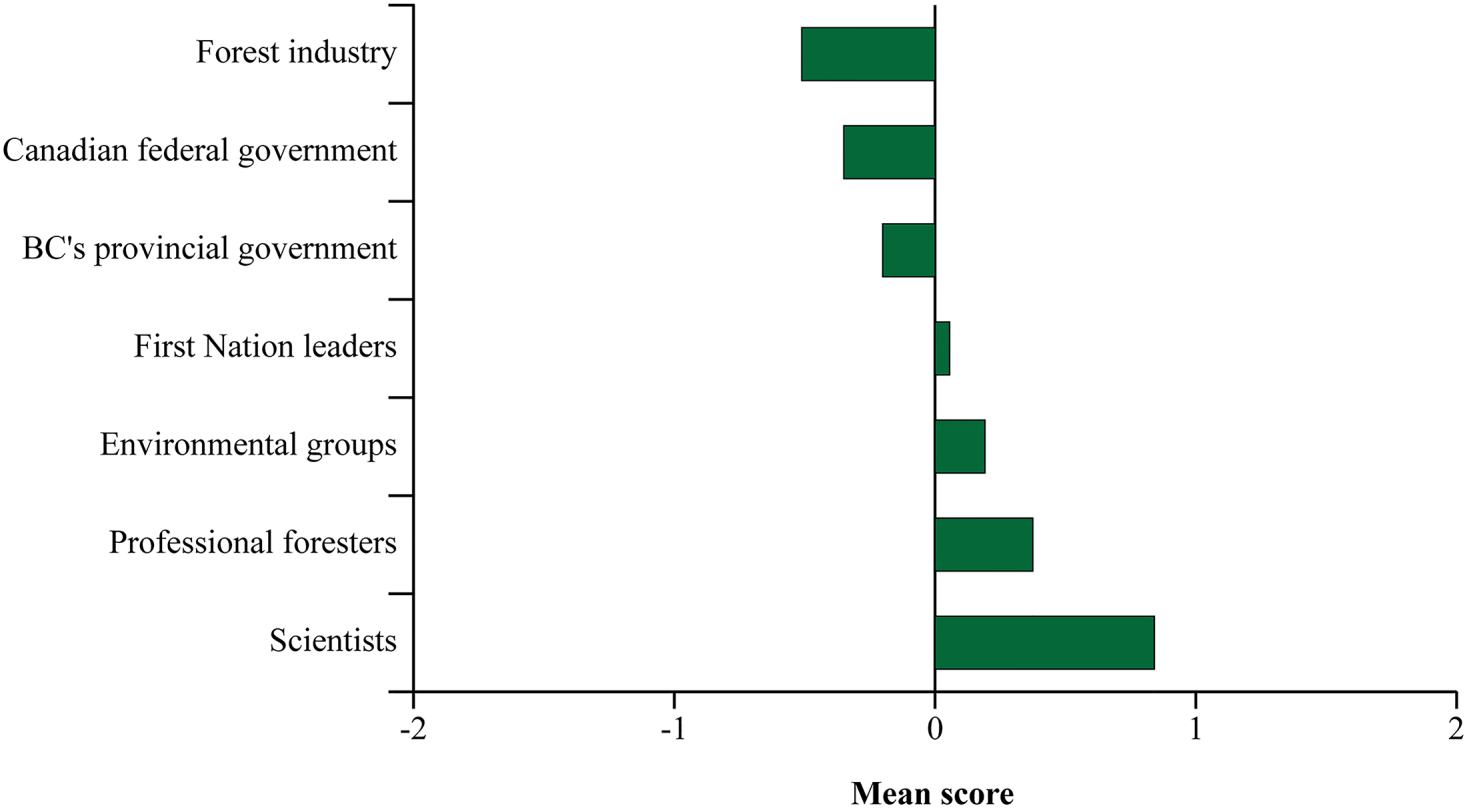
Mean scores representing the level to which respondents trust the groups of actors when it comes to providing information about climate change issues in BC’s forests, with -2 = strongly distrust and 2 = strongly trust.

Fig 2 shows respondents’ average level of trust for seven groups of actors when it comes to providing information about climate change issues in BC’s forests. A factor analysis (Table 5) showed that respondents had a different pattern of trust towards different actors, which could be clustered into three groups: 1) both levels of government and the forest industry, 2) First Nations, environmental groups and scientists, and 3) professional foresters. Nonetheless, because of their potential different influence on level of support for the different strategies, the seven individual scores were used in the regressions.

**Table 5:** Trust scale items and factor loadings. Factors’ loadings in bold indicate that they have been selected in a factor.

	Loading		
**Factor 1** (α = 0.76)			
Industry	**0.513**		0.236
BC's provincial government	**0.916**	0.110	
Canadian federal government	**0.691**	0.274	0.110
**Factor 2** (α = 0.73)			
Scientists		**0.548**	0.198
Environmental groups		**0.901**	
First Nations leaders		**0.640**	
**Factor 3**			
Professional foresters	0.285	0.170	**0.941**

The divergence in trust of different bodies observed illustrates an important divide in public opinion that can be traced back to the1980s when forests became contested spaces [11, 81]. Before then, forest policy in BC (and in Canada) was oriented towards the interests of the industry. During this time, forestry was based on the concept of sustained yield to ensure efficient exploitation of timber. However, a shift in forest management has led to the recognition of the important role played by various non-timber values, including ecological, aesthetic and cultural values [31, 38, 82]. At the same time, various groups who opposed traditional forest practices started to gain political and public influence [11]. In particular, environmental groups, through communications and marketing campaigns, have gained increased public support and influence for their conservation goals [12]. In addition, First Nations have been obtaining considerable political and judicial recognition of their rights through court decisions [68, 83]. While the interests of environmental groups and First Nations do not always align, they have regularly formed coalitions and are both often associated with the conservation movement [69].

Increased public scrutiny, combined with government reforms to BC’s forest policies (e.g., changes in forest practice codes) that have been viewed by some as unsuccessful, have led to increasing public distrust towards the historical partnership between the government and the forest industry and, ultimately, towards commercial forest management [11, 81]. In contrast, the comparatively greater level of trust in scientists and professional foresters suggests that the public is more willing to rely on expert judgement when addressing climate change issues in BC’s forests. One respondent commented: “We must rely on scientists and professional foresters and not on any level of government or industry to give the ‘straight’ facts instead of alternative facts about BC's forests and climate change”.

**Fig 3:**
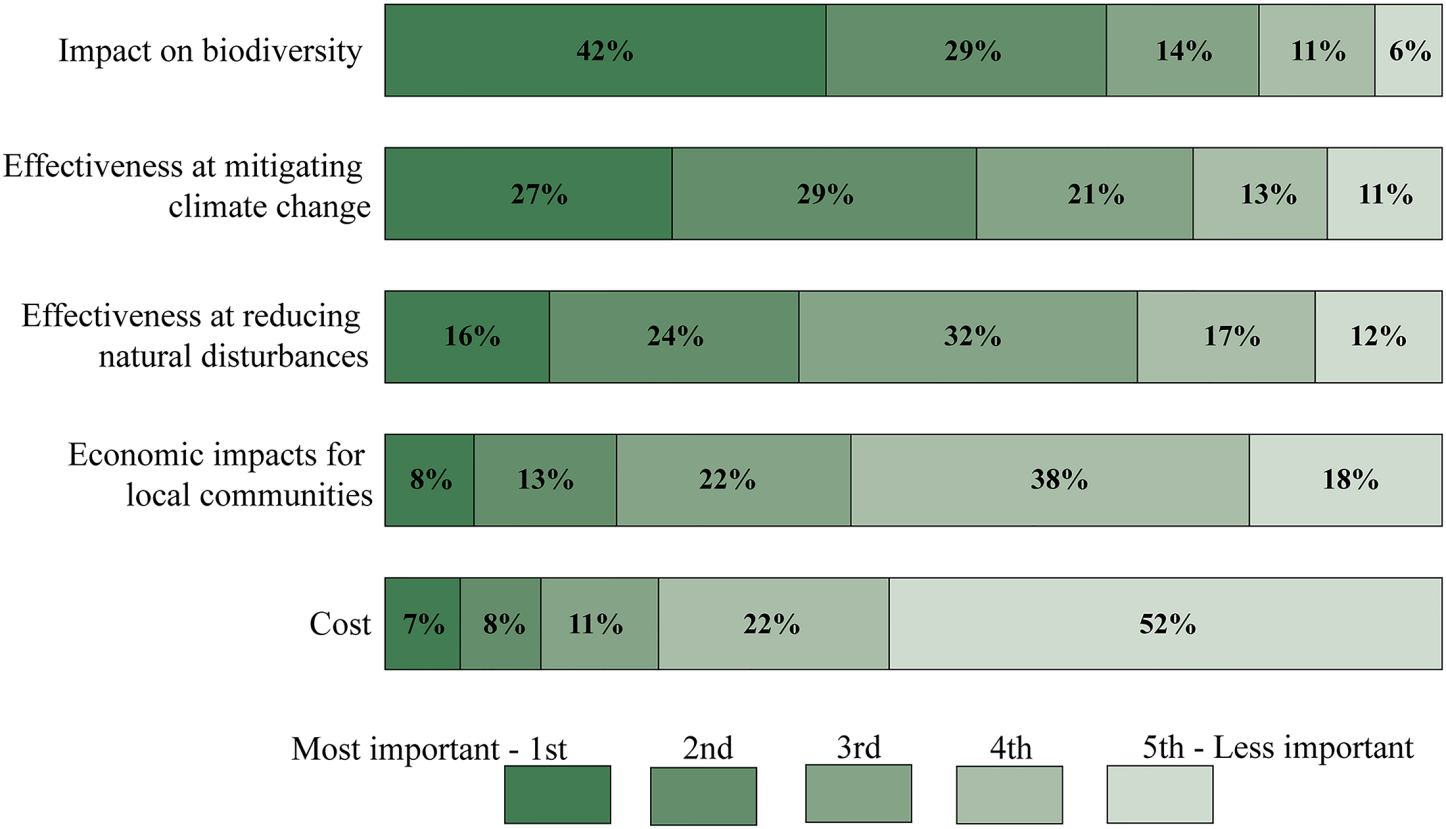
Ranking in order of relative importance (from the most to the less important) of five possible outcomes to consider when selecting forest management strategies to mitigate climate change in BC’s forests.

Most respondents identified the impact on biodiversity as the most important objective to consider when selecting mitigation strategies in BC’s forests (Fig 3), reinforcing the high prevalence of biocentric-oriented individuals in the BC population. The next highest priorities, the effectiveness at mitigating climate change and at reducing natural disturbances, signify the perceived importance of dealing with climate change and its consequences. In contrast, more than half of the respondents ranked cost as their lowest priority, followed closely by economic impacts on local communities. This result corroborates observations made by other studies in BC that show the greater appraisal of ecological forest values compared to economic values [14, 84]. The most preferred objective identified by each respondent was used as a binary variable in the multiple regressions.

### 5.2 Level of support for different mitigation strategies

**Fig 4:**
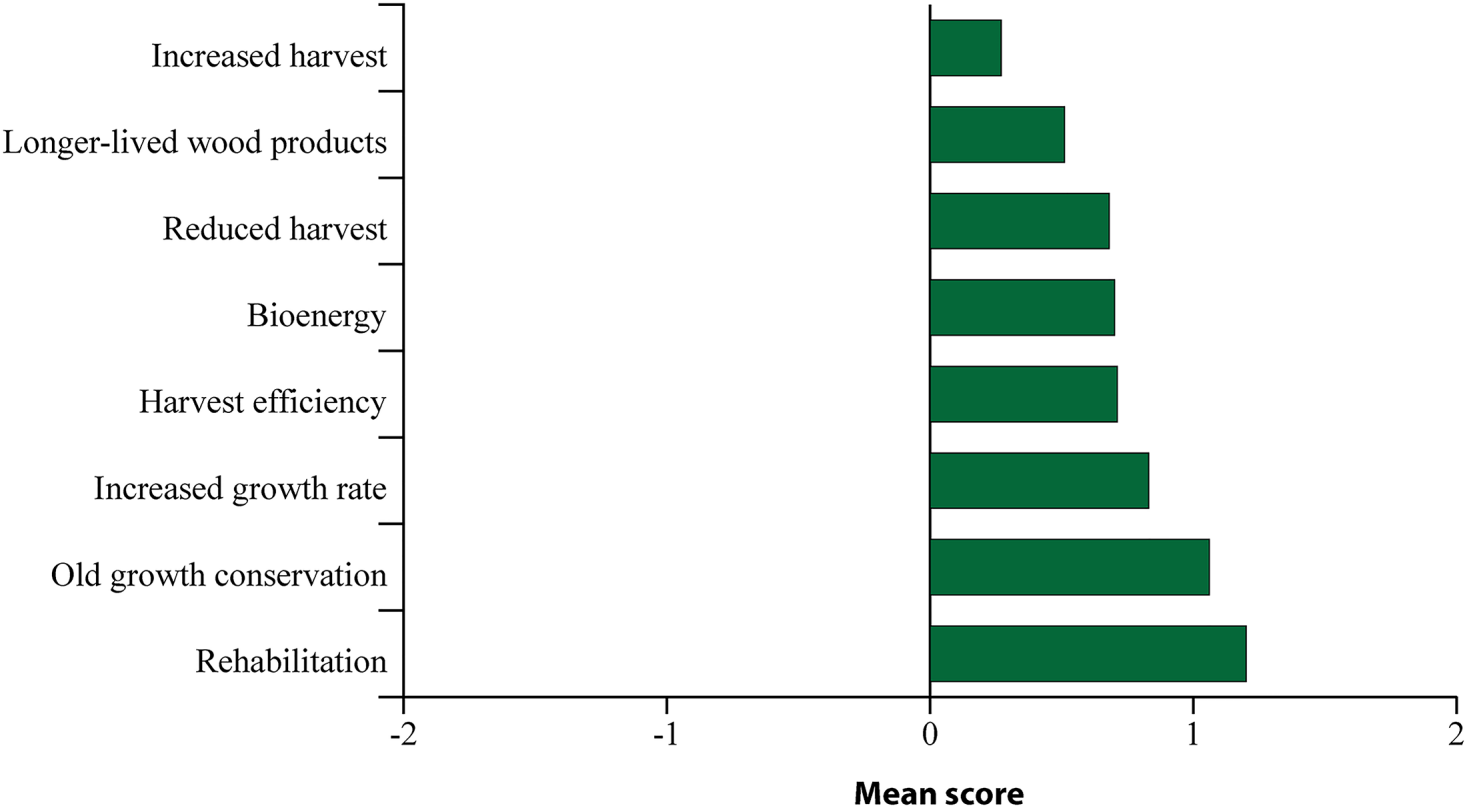
Mean scores representing the degree of support for, or opposition to the climate change mitigation strategies, with -2 = strongly oppose and 2 = strongly support.

On average, respondents supported all eight of the climate mitigation strategies presented (Fig 4). Two class of mitigation strategies emerged from a factor analysis (Table 6): factor 1 describes similar level of support for mitigation strategies oriented towards conservation (hereafter, “conservation strategies”), whereas factor 2 describes support for enhanced management and the use of harvested wood products (hereafter, “enhanced forest management strategies”). All mitigation strategies clearly loaded into one of the two factors except for rehabilitation (loading of 0.418 with conservation strategies against 0.407 with enhanced forest management strategies). Consequently, rehabilitation was not aggregated in any of the two factors and was, therefore, excluded from the regression analysis.

**Table 6:** Scale items and factor loadings of respondents’ support or opposition to climate mitigation strategies. Factors’ loadings in bold indicate that they have been selected in a factor.

	Loading	
**Factor 1** (Conservation strategies, α = 0.62)		
Reduced harvest strategy	**0.623**	–
Old growth conservation strategy	**0.703**	0.123
**Factor 2** (Enhanced forest management strategies, α = 0.66)		
Bioenergy strategy	0.225	**0.518**
Harvest efficiency strategy	0.143	**0.582**
Increased harvest strategy	-0.247	**0.542**
Increased growth rate strategy	0.193	**0.476**
Longer-lived wood product strategy	0.168	**0.515**
**Not dominant in either factor**		
Rehabilitation	0.418	0.407

A significant difference was found between the average scores of the conservation strategies (mean = 0.87, standard deviation = 0.79), enhanced forest management strategies (mean = 0.61, standard deviation = 0.57) and rehabilitation strategy (mean = 1.20, standard deviation = 0.74; F(2, 4449) = 263.4, p<0.001). *Post hoc* comparisons using the Tukey HSD test revealed that the mean scores of the three types of strategies significantly differed from each other at p < .05. Respondents generally indicated higher levels of support for conservation strategies compared to enhanced forest management strategies. Nevertheless, rehabilitation received the highest level of support with as many as 85% of respondents either supporting or strongly supporting the strategy. This result on rehabilitation suggests that the public is concerned about increased natural disasters, notably the recent mountain pine beetle epidemics [59] and forest wildfires that had devastating impacts on BC’s forests in recent years. The widespread level of support for rehabilitation implies that a majority of people in BC believe that restoration of unhealthy forests is a priority that needs to be addressed, notwithstanding how it is implemented on the ground. It also explains why rehabilitation did not have explanatory power and was excluded from the factor analysis.

### 5.3 Variation in basis of support for both categories of mitigation strategies

Multiple linear regressions were used to evaluate the effects of the independent variables on the two categories of mitigation strategies highlighted by the factor analysis, conservation strategies and enhanced forest management strategies (Table 7). The following independent variables were all entered in the regressions: risk perception of, and personal experience with, climate change; belief on the cause of climate change; knowledge of climate change in the context of forest management; environmental value (biocentric and anthropocentric); trust in seven groups of actors (separately); first prioritized outcome; age; gender; education; employment in the forest sector; political orientation; and residence type (urban/suburban/rural).

**Table 7:** Models of multiple linear regressions for each category of mitigation strategies. Bold items were found to be significant.

Independent variables	Conservation strategies	Enhanced forest management strategies
Intercept	2.34***	2.18***
Cause of CC (Mostly human activities)	0.03	0.03
Perceived risk of CC	0.11***	0.04
Experience with CC (No experience)	0.006	0.02
Knowledge of CC and forestry	-0.02	0.007
Anthropocentric	-0.11***	0.03
Biocentric	0.31***	0.12***
Outcome (cost)	-0.26**	-0.06
Outcome (CC mitigation)	-0.05	0.03
Outcome (biodiversity)	-0.02	-0.05
Outcome (economic local impact)	-0.29***	-0.02
Outcome (CC adaptation; baseline)	N/A	N/A
Trust (scientist)	0.01	0.07**
Trust (industry)	-0.11***	-0.03
Trust (federal)	-0.05	0.009
Trust (provincial)	0.04	0.02
Trust (ENGO)	0.07*	-0.02
Trust (forester)	-0.06*	0.11***
Trust (First Nations)	0.12***	-0.01
Age	0.0002	0.003**
Gender (male)	-0.04	0.06*
Education (high)	0.01	-0.08**
Employment in forest sector (employed)	-0.20*	-0.18*
Political orientation (conservative)	-0.06	0.07*
Political orientation (liberal)	0.03	0.04
Political orientation (no party; baseline)	N/A	N/A
Residence type (rural)	-0.03	-0.02
Residence type (urban)	-0.004	-0.06
Residence type (suburban; baseline)	N/A	N/A
Adjusted R^2^	0.39	0.10
F value	33.2***	6.8***

* p ≤ 0.05;

** p<0.01;

*** p<0.001

Prior to discussing the effects of the independent variables in this analysis, it is worth noting an interesting ancillary finding related to a divergence in how the two regression models fit the data and predict support. In effect, although the F-values of both models were significant, the regression model associated with conservation strategies (adjusted *R*^2^ = 0.39) better explained the variance in the data than the model for the enhanced forest management strategies (adjusted *R*^2^ = 0.10), suggesting that variables other than the ones used in the models may better explain support for enhanced forest management strategies.

Divergence in respondents’ familiarity with both categories of strategies may partly explain the contrasting predictive capacity of the models. On the one hand, most people in BC are familiar with forest conservation approaches due to their prominence in public and political debates since the 1980’s [11]. Furthermore, forests have become a major focal point in climate discussions since the mid-2000s when the United Nations Framework Convention on Climate Change (UNFCCC) began considering the mitigation potential associated with reducing tropical deforestation, a mechanism that would be ultimately known as *Reducing Emissions from Deforestation and Forest Degradation in Developing Countries* (REDD+) [85, 86]. Canadian environmental groups, exploiting this unprecedented attention given to forests, have further promoted the role of forests and their conservation in mitigating climate change [87, 88, 89]. On the other hand, the mitigation potential of forest management and the use of wood products has not received as much public attention and the effectiveness of such strategies might still be widely unrecognized by the general population. This lack of public attention for the role of forest management in mitigating climate change, combined with a general unfamiliarly [32, 59] with forestry, may explain why the enhanced forest management strategies model was weaker and more “random” in predicting support. While the variable on knowledge of climate change and forestry did not factor into the regression models, it would be interesting to test more thoroughly (e.g., quiz instead of self-reported knowledge) how variation in knowledge on specific forest management strategies affect support.

One of our main hypotheses was that respondents who are concerned about the risks and/or have personally experienced climate change would be more likely to support potential forest management strategies and accept their associated risks. However, the fact that none of the variables associated with beliefs on climate change entered in the enhanced forest management model indicates that respondents may have been even more preoccupied with the potential negative risks associated with implementation of the strategies. It is undeniable that enhanced forest management strategies are more closely related to industrial forest management, which faces criticism and opposition in certain groups of BC’s public [11]. It is conceivable that perceived risks and uncertainties of forestry, a variable that we did not include in our models, could have significant influence on predicting support for forest carbon mitigation actions, and more particularly enhanced forest management strategies. Based on our regression model, the profile of the respondents who are more likely to support enhanced forest management strategies—individuals who value nature and biodiversity (i.e., biocentric orientation), but who are also more likely to accept risks when they are placed into experts’ hand (i.e., high trust in scientists and professional foresters, low education)–is indeed suggestive of greater willingness to support policies despite risks and uncertainties.

**Fig 5:**
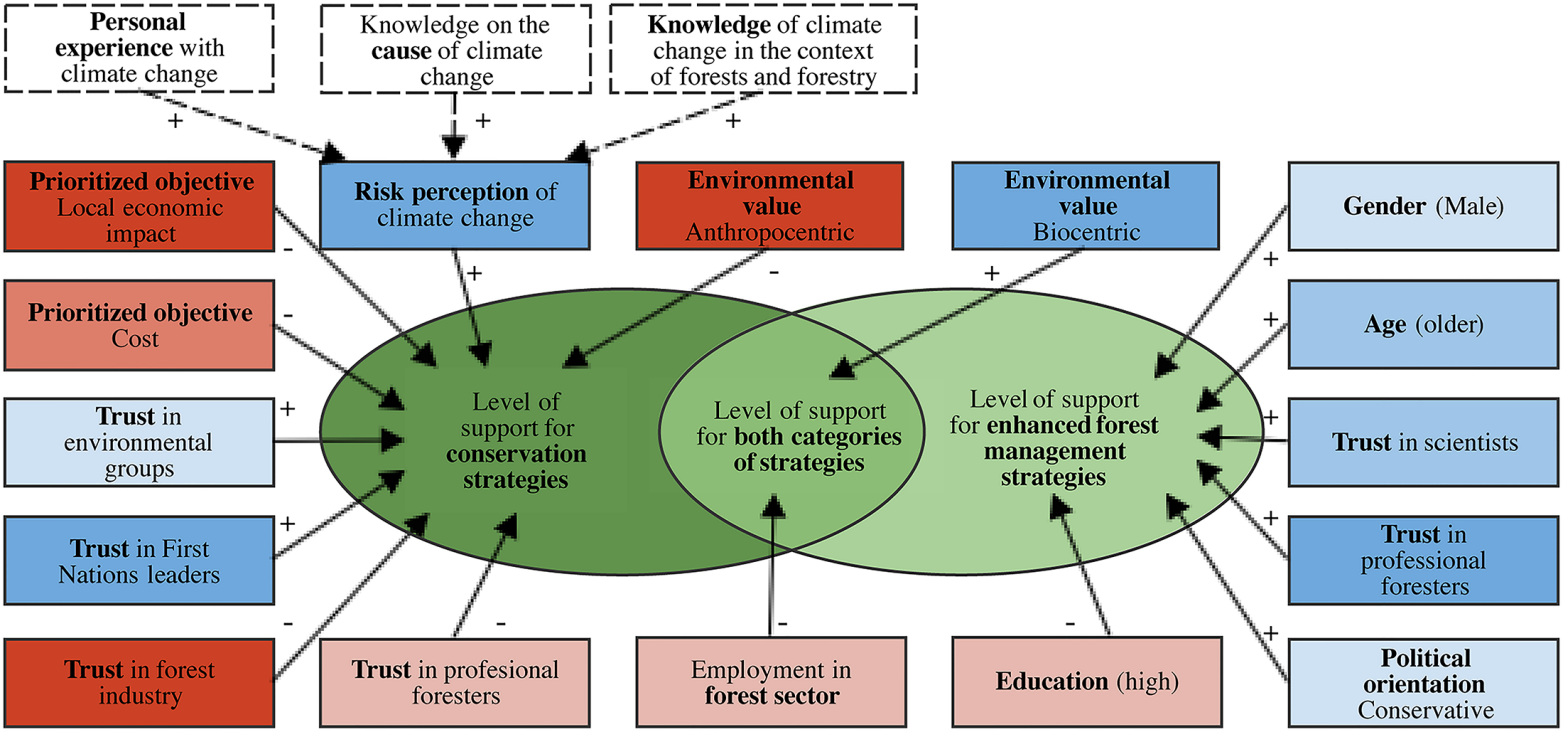
A conceptual framework highlighting the variables that affect level of support for conservation and forest management mitigation strategies. Positive (blue boxes) and negative (red boxes) signs respectively indicate positive and negative impact on level of support. Lighter (p ≤ 0.05), medium (p<0.01) and darker (p<0.001) colour shading refer to the calculated probabilities (p-values) of the variables in the linear regression. Dotted boxes identify variables that did not significantly factor into the regressions, but were correlated with risk perception of climate change.

The variables that were shown to have had an influence in our two models are illustrated as a conceptual framework in Fig 5 to highlight the key findings of the analysis. Anthropocentric-oriented individuals were less likely to support conservation strategies, confirming the assumption that such a value orientation is associated with preference for resource extraction and productive strategies over conservation [42]. In contrast, a biocentric orientation was associated with support for both categories of mitigation, a result which is somewhat counterintuitive considering that biocentric-oriented individuals normally support conservation (e.g., protection of wildlife and old growth) and oppose more intrusive forest management strategies [38]. Nonetheless, as Steel et al. [39] explain, “these value orientations [biocentric and anthropocentric] are not mutually exclusive (except perhaps in their most extreme forms) and are multidimensional […] Somewhere in between the two ends one gets a mixture of the orientations”. Furthermore, as hypothesized at the outset, the increasing prominence of climate change in any environmental policy debate may be compelling biocentric-oriented individuals to (re)consider more human-intensive interventions when they believe in their potential effectiveness to reduce greenhouse gases emissions and, hence, the impacts of climate change on forests [43].

Our results also highlight how trust in decision-makers and other actors diverges and can influence level of support for different strategies. Trust in the forest industry and professional foresters had a negative impact on the level of support for conservation strategies, whereas trust in environmental groups and First Nations leaders had the opposite effect, emphasizing, once again, the divide in public opinion when discussing industrial forest management and conservation in BC. In contrast, the associations found between confidence in scientists and professional foresters and support for enhanced forest management strategies confirm that individuals’ trust in these two groups increases the likelihood of supporting potential forest management practices [55, 66]. Experts will have an important role to play in highlighting and disseminating the potential and limitations of different mitigation strategies and in shaping public opinions on, and approval of, future forest carbon mitigation policies.

A high perceived risk from climate change was associated with support for conservation strategies, affirming the prevailing assumption that individuals are more likely to take actions and support policies when they perceive that an environmental issue will negatively affect them [45]. While knowledge of climate change in the contexts of forests and forestry did not enter into either of the two regression models, it was found to be positively correlated with risk perception of climate change (Pearson’s r = 0.25, p<0.001). That being said, the low strength of association (i.e., small correlation coefficient) between the two variable seems to corroborate the conclusion that an individual’s knowledge is a poor predictor of their risk perception, and that other demographic, contextual and normative factors have more influence (reviewed by McFarlane et al., [55]). However, it is important to acknowledge that the use of self-reported knowledge may limit our capacity to test this hypothesis.

In contrast, significant differences in the risk perception scores were found between respondents who believe climate change is mostly caused by human activities (mean = 3.76, standard deviation = 0.65) and those who did not (mean = 3.00, standard deviation = 0.80; t(1229) = -18.78, p<0.001), as well as between respondents that experienced events that they associate with climate change (mean = 3.66, standard deviation = 0.71) and those that did not (mean = 3.09, standard deviation = 0.83); t(1232) = 13.43, p<0.001). This confirms the assumptions that belief in the human causes of, and personal experiences with, climate change directly affect risk perception [62]. Respondents who endorsed cost and economic local impact as their highest priority generally had lesser levels of support for conservation strategies, which was also observed in other studies exploring how values affect forest management preferences [39, 90].

Individuals who indicated being either directly or indirectly employed by BC’s forest sector were less likely to support both categories of strategies. This result is somewhat counterintuitive considering that some enhanced forest management strategies (e.g., bioenergy and longer-lived wood products) could arguably increase employment opportunities in the forest sector (see Xu et al. [15] for socioeconomic impacts of various forest carbon mitigation strategies in BC). On the other hand, BC’s forest sector has recently faced an economic downturn that led to a drop of 40% in employment between 2003 and 2011 [91]. Even though the forest sector has slowly been recuperating (i.e., employment increased by 17.6% since 2015), the recent uncertain economic climate might explain why forest sector employees prefer the *status quo* over the implementation of new forest management strategies with potentially uncertain economic impacts.

Finally, a conservative political orientation has a positive effect on the level of support for enhanced forest management strategies. In contrast, gender (male) and age (older individuals) have positive impacts on levels of trust for enhanced forest management strategies, whereas higher educated individuals were less likely to support these strategies. As in other studies [33, 39, 92], our results indicate that, on average, men (mean = 2.16, standard deviation = 0.87) and conservative individuals (mean = 2.23, standard deviation = 0.91) have respectively greater anthropocentric orientation than women (mean = 1.97, standard deviation = 0.85; t(1471) = -4.27, p<0.001) and liberal individuals (mean = 1.85, standard deviation = 0.80; t(805) = 6.51, p<0.001). Higher age and lower education is also often correlated with anthropocentric belief (idem), although no significant effect was found for these two variables in this study. As discussed elsewhere, a greater anthropocentric orientation may explain, at least partly, a lesser preference for conservation strategies. Men and older individuals have also been positively associated with lower perceived risks from forestry activity [92], which can also explain why they are more likely to support enhanced forest management strategies.

## 6 Conclusions and policy implications

In this study, we examined public levels of support for forest-based carbon mitigation strategies in British Columbia. Overall, respondents ascribed a high level of importance to forest carbon mitigation and supported all of the eight proposed strategies, indicating that the BC public is inclined to consider alternative practices in managing forests and wood products to mitigate climate change. That said, differences were found in levels of support for the mitigation strategies. In general, we found greater levels of support for a rehabilitation strategy, and to a lesser extent for conservation-focused strategies over enhanced forest management strategies. We also highlighted how demographics and explanatory variables extracted from the literature on environmental and climate risk perception appear to play a role in predicting levels of support for conservation and/or enhanced forest management strategies.

In the last decade, at least two different views have emerged from discussions on forest carbon mitigation that mirror, in many ways, the public divide observed in the 1980s. On the one hand, environmental groups have used the momentum around international efforts at reducing emissions from deforestation to push forward their conservation agenda. On the other hand, members of the forest carbon scientific community fear that criticisms made by the environmental community towards forest management practices may potentially exacerbate the “misunderstanding by the public that sustainable management, of which logging is a part, contributes to climate change” [93].

Nevertheless, the discourses between the two sides are increasingly converging, especially in light of the recent observed impacts that climate change is exerting on BC’s forests (e.g., increased number and intensity of forest fires and insect epidemics). For instance, in 2010, a group of environmental organizations and worker unions published a paper that “advocates for a broad approach to managing our publicly-owned forest resources. It invites us to re-imagine forestry in BC, not through the traditional (and opposing) lenses of either maximizing human use, or maximizing protected areas, but rather, with a view towards maximizing carbon storage” [94]. While points of discord persist, an opportunity exists to initiate discussions and joint actions towards a set of comprehensive, consensus-based forest carbon mitigation policies in the province. By showing that the public is generally positively predisposed towards forest carbon mitigation, results of this study reinforce this opportunity.

In addition, notwithstanding potential divergences in preferences for other types of strategies, it is clear that the rehabilitation of areas recently affected by insects and fires is widely supported by BC’s general public. The recent creation of the Forest Carbon Initiative announced by the BC government in its Climate Leadership Plan, which will focus on rehabilitating Mountain Pine Beetle and wildfire impacted sites [6], is, therefore, timely and should be well received by the public. Nevertheless, while the rehabilitation of such areas will definitely provide mitigation benefits, recent studies indicate that no single strategy will be able to maximize forest carbon mitigation alone; only an approach combining various practices and accounting for regionally-differentiated ecological and socio-economic features can take full advantage of BC forests’ mitigation potential [15, 16, 19]. Furthermore, various reforestation strategies that explicitly take climate change into account, such as assisted migration and assisted gene flow, can be used to rehabilitate BC’s public forests [43, 95, 96]. Further research is needed to evaluate public preferences regarding such approaches.

Finally, our results indicate and reinforce known critical issues, particularly in terms of public trust towards government decision-makers. One way for the government to address this issue of trust would be to secure partnerships with other influential and trusted actors such as environmental organizations, First Nations leaders, and scientists. Furthermore, it will be imperative to ensure active participation of the public, First Nations, and all stakeholders for the credibility and legitimacy of decision-making processes and their outcomes, especially considering that the issues of forest management and climate change are complex and scientific in nature [97, 98]. Further research is also needed to evaluate public opinion on how forest carbon mitigation strategies should be implemented through actual policies (e.g., voluntary carbon offsets program, changes in forest management policies and practices, climate change policies) and how the trade-offs associated with the implementation of different mitigation strategies (e.g., effectiveness at mitigating climate change vs. environmental, social and economic impacts) further affect public preferences.

## Supporting information

S1 Appendix Overview of survey questions. (DOCX)

S2 Appendix Overview of survey respondents’ demographic. (DOCX)
