# Supplementary material for: Public perceptions about climate change mitigation in British Columbia's forest sector
Source: PLoS One. 2018 Apr 23;13(4):e0195999. doi: 10.1371/journal.pone.0195999 (PMC5912731; doi:10.1371/journal.pone.0195999)
Supplement: S1 Appendix — (DOCX) [file pone.0195999.s001.docx]

**S1 Appendix.** Overview of survey questions

This appendix provides the complete questions used in the online survey to assess the dependent and independent variables.

**DEPENDENT VARIABLES**

1. **Level of support for mitigation strategies**

*Q. To what degree would you support or oppose the following climate change mitigation strategy?*

*a) Bioenergy strategy; b) Harvest efficiency strategy; c) Reduced harvest strategy; d) Increased harvest strategy; e) Increased growth rate strategy; f) Longer-lived wood product strategy; g) Old growth conservation strategy; h) Rehabilitation strategy*

Measurement scale: 1=Strongly oppose; 5=Strongly support

**INDEPENDENT VARIABLES**

1. ***Belief on the cause of climate change***

*Q. Do you think that climate change is...*

Measurement scale: 1) Caused mostly by human activities; 2) Caused mostly by natural changes in the environment; 3) Caused equally by natural changes in the environment and human activities; 4) Neither, because climate change isn’t happening; 5) Other, please specify; 6) I don’t know

1. Perceived risk of climate change

*Q. Overall, how concerned are you about climate change?*

Measurement scale: 1=Not concerned at all; 5=Extremely concerned

*Q. How often do you worry about the potentially negative consequences of climate change?*

Measurement scale: 1=Never; 5=always

*Q. To what extent do you that climate change will harm...*

*a) you personally?; b) People in BC?; c) BC’s forests?; d) the world population?; e) the world’s forests?*

Measurement scale: 1=Not at all; 5=extensively

1. Personal experience with climate change

*Q. In the last 10 years, how often (in total) have you personally experienced any type of natural disaster (e.g., wildfire, flood) or extreme weather event (e.g., severe heat waves, storms, droughts) in your local BC area that you associate with climate change?*

Measurement scale: 1) Never; 2) Once; 3) Twice; 4) Three times; 5) Four times or more; 6) I don't know

1. Perceived importance of climate change mitigation

*Q. In BC, how important do you think it is to deal with...*

*a) Climate change mitigation across all sectors?; b) Climate change mitigation in the forest sector specifically?*

Measurement scale: 1=Not important at all; 5=Extremely important

1. Knowledge on climate change and forest management

*Q. How knowledgeable are you about the following topics?*

*a) The role of forests in regulating climate by reducing greenhouse gas emissions and increasing carbon removals from the atmosphere; 2) The potential consequences of climate change on BC's forests; 3) The current forest management strategies implemented in BC's forests; 4) Strategies involving BC’s forests to reduce climate change.*

Measurement scale: 1 = Not at all knowledgeable; 5 = Very knowledgeable

1. Environmental values orientation

*Q. To what extent do you agree or disagree with the following statement?*

*a) Forests should be managed to meet the needs of as many people as possible; b) The primary use of forests should be for products that are useful to humans; c) Forests that are not used for the benefit of humans are a waste of our natural resources; d) Forests are valuable only if they produce jobs and income for people; e) Forests give us a sense of peace and well-being; f) Whether or not I get to visit the forest as much as I like, it is important for me to know that forests exist in BC; g) Forests have a right to exist for their own sake, regardless of human concerns and uses; h) Humans should have more love, respect and admiration for forests.*

Measurement scale: 1=Strongly disagree; 5=Strongly agree

1. Level of trust towards different actors

*Q. In general, to what extent do you trust the following groups when it comes to providing information about climate change issues in BC’s forests?*

*a) Scientists; b) Forest industry; c) BC's provincial government; d) Canadian federal government; e) Environmental groups; f) Professional foresters; g) First Nations leaders*

Measurement scale: -2=Strongly distrust; 2=Strongly trust

1. Objectives when selecting forest management strategies

*Q. Which of the following objectives are the most important when selecting forest management strategies to mitigate climate change in BC's forests?  Please rank the objectives in order of importance to you personally.*

*a) Their cost; b) Their effectiveness at mitigating climate change; c) Their impacts on biodiversity (for example, trees, plants and animals); d) Their effectiveness at reducing natural disturbances (for example, fires and insects); e) Their economic impacts for local communities.*

Measurement scale: 5=1^st^ (most important); 4=2^nd^; 3=3^rd^; 2=4^th^; 1=5th

1. Age

*Q. What is your age?*

Measurement scale: Numeric value 19+

1. Gender

*Q. What is your gender?*

Measurement scale: a) Male; b) Female; c) Prefer not to answer

1. Education

*Q. What is the highest level of education you have completed?*

Measurement scale: a) Elementary school, b) High school, c) Vocational/technical school, d) Some university; e) Bachelor degree (or equivalent), f) postgraduate degree; g) Prefer not to answer

1. Employed by forest sector

*Q. Are you or one of your immediate family members employed in British Columbia’s forest sector?*

Measurement scale: a) Yes, directly employed; b) Yes, indirectly employed (supplying goods and services to the forest sector; c) No; d) Prefer not to answer

1. Political orientation

*Q. Generally speaking, which provincial political party do you most closely associate yourself with?*

Measurement scale: a) British Columbia Liberal Party; b) British Columbia New Democratic Party; c) British Columbia Conservative Party; d) Green Party of British Columbia; e) Other, Please Specify; f) I don’t associate myself with any Party; g) Prefer not to answer;

1. Residence type

*Q. Which of the following best describes the area you live in?*

Measurement scale: a) Urban; b) Suburban; c) Rural; d) Prefer Not to Answer
