# Supplementary material for: Public perceptions about climate change mitigation in British Columbia's forest sector
Source: PLoS One. 2018 Apr 23;13(4):e0195999. doi: 10.1371/journal.pone.0195999 (PMC5912731; doi:10.1371/journal.pone.0195999)
Supplement: S2 Appendix — (DOCX) [file pone.0195999.s002.docx]

**S2 Appendix.** Overview of survey respondents’ demographic

| **Variable** | **#** | **Percentage** |
| --- | --- | --- |
| **Age**  19-34 years old  35-54 years old  ≥ 55 years old  Prefer not to answer | 391  544  548  1 | 26.3%  36.7%  36.9%  0.1% |
| **Gender**  Male  Female  Other | 710  768  6 | 47.8%  51.8%  0.4% |
| **Education**  Low  Elementary school  High school  Vocational/technical school  High  Some university/college  Bachelor’s degree  Graduate degree  Prefer not to answer | 12  352  362  35  458  244  22 | 0.7%  23.7%  24.4%  2.4%  30.9%  16.4%  1.5% |
| **Employment in forest sector**  Employed  Directly  Indirectly  Not employed  Prefer not to answer | 54  20  1398  12 | 3.6%  1.3%  94.2%  0.8% |
| **Political orientation**  Conservative  BC Conservative Party  BC Liberal Party  Liberal  BC New Democratic Party  Green Party of BC  I don’t associate myself with any Party  Prefer not to answer | 105  332  274  97  521  155 | 7.1%  22.4%  18.5%  6.5  35.1%  10.4% |
| **Region of residence**  Urban  Suburban  Rural  Prefer not to answer | 729  581  154  20 | 49.1%  39.2%  10.4%  1.3% |
